# Overexpression a “fruit-weight 2.2-like” gene *OsFWL5* improves rice resistance

**DOI:** 10.1186/s12284-019-0315-9

**Published:** 2019-07-16

**Authors:** Bei Li, Shengyuan Sun, Xianmin Gao, Mengxiao Wu, Yong Deng, Qinglu Zhang, Xianghua Li, Jinghua Xiao, Yinggen Ke, Shiping Wang

**Affiliations:** 1 National Key Laboratory of Crop Genetic Improvement, National Center of Plant Gene Research (Wuhan), Huazhong Agricultural University, Wuhan, 430070 China; 2 Jiangsu Key Laboratory of Crop Genetics and Physiology/Co-Innovation Center for Modern Production Technology of Grain Crops, Key Laboratory of Plant Functional Genomics of the Ministry of Education, Yangzhou University, Yangzhou, 225009 China

## Abstract

**Background:**

Rice (*Oryza sativa*) feeds half of the world’s population. Rice grain yield and quality which are constrained by diseases and mineral nutritions have important human healthy impacts. Plant “fruit-weight 2.2-like” (*FWL*) genes play key roles in modulating plant fruit weight, organ size and iron distribution. Previous work has uncovered that the grains of *OsFWL5-*oeverexpressing rice accumulated more beneficial element zinc (Zn) and less toxic element cadmium (Cd) content. However, whether *FWL* genes play roles in rice resistance remains unknown.

**Findings:**

Here, we validated that one of rice *FWL* genes *OsFWL5* plays a positive role in defense to *Xanthomonas oryzae* pv. *oryzae* (*Xoo*). Overexpresion of *OsFWL5* promotes H_2_O_2_ accumulation and cell death. The *OsFWL5-*overexpresing plants show activated flg22-induced reactive oxygen species (ROS) generation, and increased resistance to *Xoo*, indicating that *OsFWL5* functions to increase pathogen-associated molecular pattern (PAMP)-triggered immunity in rice. The activated defense response is associated with increased the expression of genes involved in jasmonic acid (JA)-related signaling. Furthermore, Cd can induce rice resistance to *Xoo*, and *OsFWL5* is required for Cd-induced rice defense response.

**Conclusion:**

Putting our finds and previous work together, *OsFWL5* could be a candiate gene for breeders to genetically improve rice resistance and grain quality.

**Electronic supplementary material:**

The online version of this article (10.1186/s12284-019-0315-9) contains supplementary material, which is available to authorized users.

## Findings

Mineral nutrients and diseases constrant crop production and quality. To increase crop yields, tromendous fertilizers and pesticide have been used resulting in adverse impacts on environment (Withers and Lord, 2002; Niño-Liu et al., 2006). Beside up take essential mineral nutrients (e.g. nitrogen, Zn) for orchestrating development and defense response, plants also take up non-essential and toxic elements (e.g. Cd and arsenic) which induce chronic and toxic effects in humans (White and Broadley, 2009; Zhao et al., 2010; Clemens and Ma, 2016). As it feeds about half of the world’s population, rice (*Oryza sativa*) grian quality is fundamental importance for human health. Thus, applying genetic approaches to improve rice plant resistance, to increase the accumulation of essential nutrients, and to reduce the concentration of chronic and toxic elements in grains have very important agricultral and human healthy impacts.

Tomato *FW2.2* was identified as a key to control fruit weight and size (Frary et al., 2000). Plenty of findings imply that FW2.2-like proteins play various roles in plant. *Arabidopsis FWL* genes *plant cadmium resistance 1* (*AtPCR1*) involved in cadmium resistance (Song et al., 2004), *AtMCA1* and *AtMCA2* were found to mediate Ca^2+^ uptake (Yamanaka et al., 2010). Soybean *FWL* gene *GmFWL1* was found to affect the nodule organogenesis in plant interaction with the nitrogen-fixing symbiotic bacterium *Bradyrhizobium japonicum* (Libault et al., 2010). Overexpression of *OsFWL5*/*OsPCR1* increases rice grain Zn content and reduces Cd content (Song et al., 2015). However, no *FWL* gene was designated to be associated with defense response so far.

Bacterial blight caused by *Xoo* is one of the most devastating bacterial diseases of rice worldwide. To demonstrate whether *OsFWL5* involving in rice resistance to *Xoo*, we first checked the expressional patterns of *OsFWL5* in rice resistant and susceptible interaction with *Xoo* strain PXO341. MKbZH1 carried a transgenic major disease resistance gene *Xa3/Xa26* in the genetic background of japonica/geng variety Zhonghua 11 (ZH11) conferring race-specific resistance to *Xoo* including to strain PXO341, wild type ZH11 is susceptible to *Xoo* strain PXO341 (Cao et al., 2007; Gao et al., 2010; Li et al., 2012). *OsFWL5* showed differential expression patterns in rice resistant and susceptible interactions (Additional file 1: Figure S1). The transcript level of *OsFWL5* was lower in MKbFZH1 relative to wild type before *Xoo* inoculation, while higher transcript level of *OsFWL5* was observed in resistant plants than in susceptible plants at 4, 8, 24, 48 and 72 h after *Xoo* infection. The differential expression patterns of *OsFWL5* in susceptible and resistant response in the same genetic background indicated that *OsFWL5* might be involved in the rice-*Xoo* interaction.

**Fig. 1:**
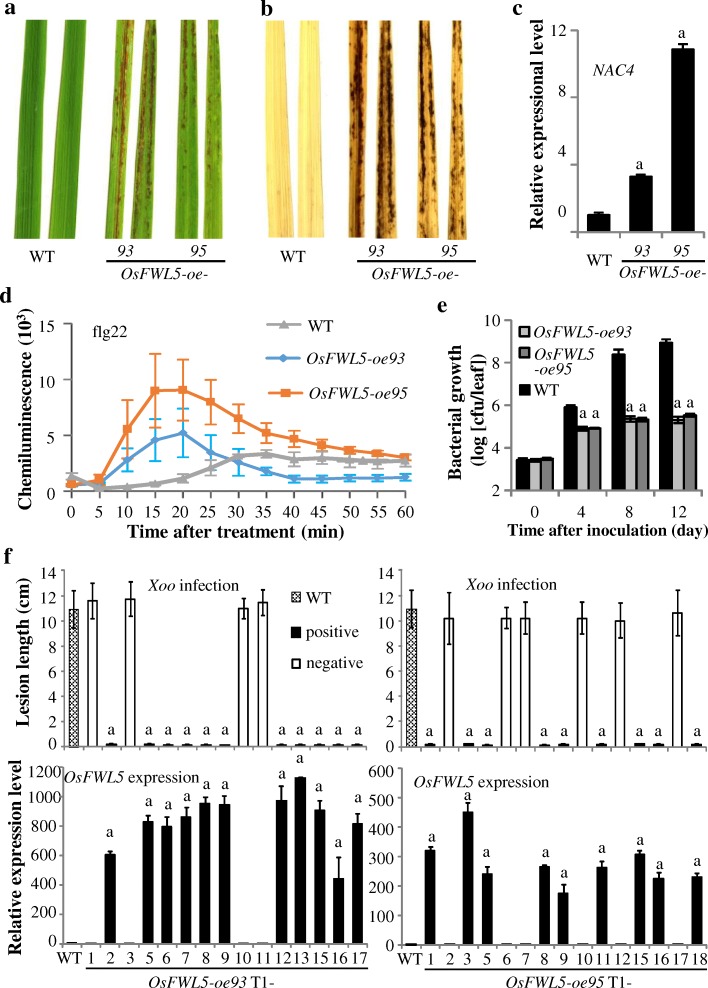
Performance of *OsFWL5-oe* plants. The “a” above bars indicate significant differences compared to wild type (WT) at *P* < 0.01. Primers and methods are listed in Additional files 2 and 3. **a** Lesion mimic phenotype of 8-week-old *OsFWL5-oe* plants. **b** DAB staining of H_2_O_2_ accumulation in 8-week-old *OsFWL5-oe* plants leaves. **c** NAC4 gene expression analysis in *OsFWL5-oe* plants leaves. Data are means ± SD (*n* = 3). **d** Flg22-induced ROS burst in the *OsFWL5-oe* and WT plants. Rice leaf disks were treated with 1 μM flg22 and water. ROS were detected with a luminol-chemiluminescence assay. Data are means ± SD (*n* = 3). **e** Growth of *Xoo* strain PXO341 on the leaves of *OsFWL5-oe* plants. Data are means ± SD (*n* = 3). cfu, colony-forming units. **f** Increased resistance of *OsFWL5-oe* plants to *Xoo* strain PXO341 was associated with increased *OsFWL5* expression. Data are means ± SD (n = 3 for gene expression, and 3 to 5 for lesion length)

We then generated *OsFWL5*-overexpressing plants (*OsFWL5*-*oe*) by transforming ZH11 with *OsFWL5* cDNA under the control of maize *ubiquitin* (*Ubi*) promoter. The *OsFWL5-oe* plants displayed a spontaneous lesion mimic (LMM) phenotype from seedling stage, and developed more serious LMM at adult stage (Fig. 1a). Many LMM show an accumulation of reactive oxygen species ROS (including H_2_O_2_) in and around lesions (Lorrain et al., 2003). To test whether the lesions of *OsFWL5-oe* plants accumulate H_2_O_2_, we stained the leaves of *OsFWL5-oe* plants with diaminobenzidine (DAB) revealing a strong accumulation of H_2_O_2_ in the *OsFWL5-oe* plants relative to WT (Fig. 1b). The appearance of LMM in *OsFWL5-oe* plants promotes us to check the expression of cell death related gene. Rice *NAC4* (a plant-specific transcription factor) positively regulates programed cell death (PCD) and activation of *NAC4* expression promotes PCD (Kaneda et al., 2009). The expression of *NAC4* were up-regulated in *OsFWL5-oe* plants (Fig. 1c). These results indicate that overexpression of *OsFWL5* promotes H_2_O_2_ accumulation and cell death.

Upon pathogen infection, the recognition of PAMPs by the pattern recognition receptors (PRRs) triggers PAMP-triggered immunity (PTI) and includes the accumulation of ROS (Jones and Dangl, 2006). Rice cells can recognize bacterial pathogen PAMP elicitor flg22 through the PRR FLS2 (Takai et al., 2008). Mutations resulting in constitutive expression of defense mechanisms cause spontaneous lesions. To examine whether overexpression of *OsFWL5* affects ROS production after PAMP elicitor flg22 treatment, we collected leaves from the *OsFWL5-oe* and WT plants and measured the ROS level after flg22 treatment using a ROS inhibition assay (Schwacke and Hager, 1992). Tissues of 4-week-old rice leaves exhibited a ROS burst when they were exposed to flg22 (Fig. 1d). In *OsFWL5-oe* plants, the flg22-induced ROS generation was earlier and higher than that in WT. These data suggested that overexpressing *OsFWL5* enhances rice PAMP-triggered immune response.

We further inoculated *OsFWL5-oe* plants with *Xoo* strain PXO341 at the booting (panicle development) stage. The *OsFWL5-oe* plants showed increased resistance to *Xoo* strain PXO341 compared to WT plants (Fig. 1e; 1f), with the lesion length ~ 0.5 cm for *OsFWL5-oe* transgenic positive plants versus ~ 11.0 cm for negative transgenic plants and WT. The increased resistance of *OsFWL5-oe* plants co-segregated with increased *OsFWL5* transcripts. The correlations between length and *OsFWL5* transcripts were − 0.926 (significant at α = 0.01; *n* = 15) and − 8993 (significant at α = 0.01; *n* = 15) for *OsFWL5-oe93* and *OsFWL5-oe95* families, respectively. Bacterial growth analysis showed that the growth rate of PXO341 on transgenic plants was significantly lower than the growth rate on WT plants at 4–12 days after infection. These results suggest that the increased resistance of the transgenic plants may be attributable to the increased expression level of *OsFWL5*.

To further investigate the role of *OsFWL5* in rice-*Xoo* interaction, we generated *OsFWL5*-knockout mutants *osfwl5* using CRISPR/Cas9 editing in ZH11. We selected two 20-nt sequences as target sites for Cas9 cleavage with one in the 5′ UTR and another one in the first exon of *OsFWL5* gene (Additional file 1: Figure S2). We found two mutant lines *osfwl5–1* and *osfwl5–2*. *osfwl5–1* carries a 242-base fragment deletion in 5′ UTR and one-base insertion in site 2; *osfwl5–2* carries a 678-base fragment deletion from site 2 to 5′ UTR of *OsFWL5* gene (Additional file 1: Figure S2). We inoculated *osfwl5* lines with *Xoo* strain PXO341 at booting stage. *osfwl5* lines developed similar lesion length as WT (Additional file 1: Figure S3a), indicating that *OsFWL5* is not necessary for *Xoo* resistance in rice. Together with the results from the above analysis, these data suggested that *OsFWL5* contributes to rice resistance by activating rice basal defense.

**Fig. 2:**
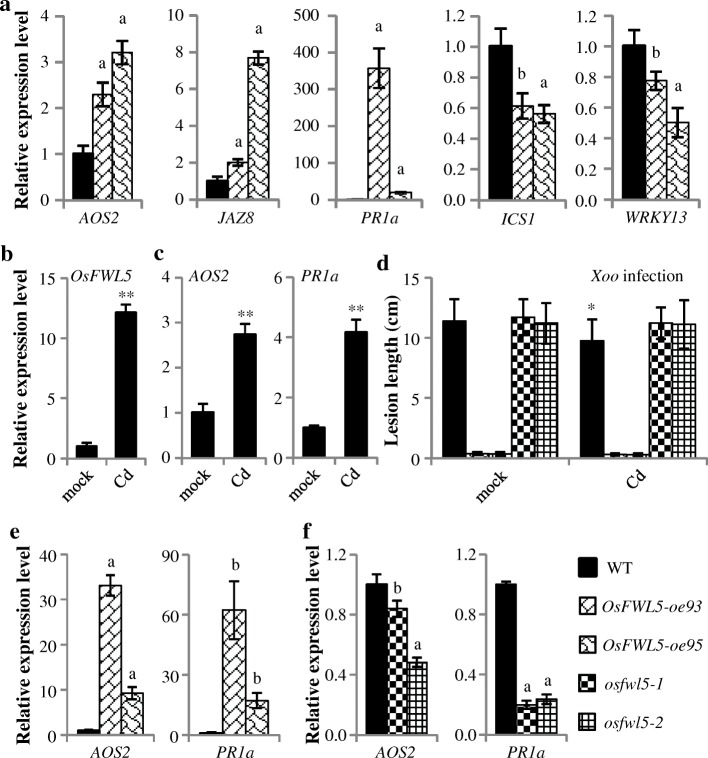
*OsFWL5* affects a set of Pathogenesis-related genes expression and rice response to Cd. ** and * indicate significant differences between Cd treatment and mock treatment at *P* < 0.01 and *P* < 0.05, respectively. The “a” and “b” above bars indicate significant differences compared to wild type (WT) at *P* < 0.01 and *P* < 0.05, respectively. Data are means ± SD (*n* = 3 for gene expression, and 5 to 15 for lesion length). Primers and methods are listed in additional files 2 and 3. **a** *OsFWL5-oe* plants accumulate more JA signaling involved genes *AOS2*, *JAZ8* and *PR1a* transcripts, and less SA signaling involved genes *ICS1* and *WRKY13* transcripts. **b** *OsFWL5* expression was induced by Cd treatment. **c** *AOS2* and *PR1a* expression was induced by Cd treatment. **d** Disease resistance analysis after Cd treatment. *OsFWL5-oe* plants accumulate more *AOS2* and *PR1a* transcripts (**e**), while *osfwl5* plants accumulate less *AOS2* and *PR1a* transcripts (**f**) after Cd treatment

The enhanced resistance of *OsFWL5-oe* plants promoted us to check the expression of defense-related genes to dissect possible defense pathways mediated by *OsFWL5. AOS2* (allene oxide synthase 2; AY062258) is involved in JA biosynthesis, *JAZ8* (jasmonate ZIM-domain protein; XP_015612402) associates with the JA-dependent signaling pathway (Mei et al., 2006; Ke et al., 2014), *WRKY13* antagonistically regulates salicylic acid (SA)- and JA-dependent signal pathway acting as a positive regulator in SA-dependent and a negative regulator in JA-dependent signal pathway, *ICS1* (isochorismate synthase 1, AK120689) is involved in SA biosynthesis (Qiu et al., 2007), *PR1a* (for acidic pathogenesis-related protein 1; AJ278436) is a SA and JA responsive gene (Ke et al.*,* 2014). The expression levels of *AOS2*, *JAZ8* and *PR1a* were significantly higher in *OsFWL5-oe* plants than those in WT (Fig. 2a). By contrast, the expression levels of *WRKY13* and *ICS1* were significantly lower in *OsFWL5-oe* plants than those in WT (Fig. 2a). We also checked the expression of these genes in *osfwl5* mutants plants, and results showed that *osfwl5* mutants plants accumulate similar *AOS2*, *JAZ8*, *PR1a* and *ICS1* transcripts, and slightly more *WRKY13* transcripts relative to wild type (Additional file 1: Figure S3b). These data indicated that overexpression of *OsFWL5* promotes defense response associated with activated JA-dependent pathway but repressed SA-dependent pathway.

As *OsFWL5* is involved in grain Cd distribution (Song et al., 2015), we treated wild type ZH11 with Cd to analyze *OsFWL5* expression. Result showed that Cd treated plants accumulated more *OsFWL5* transcripts than mock treated plants did (Fig. 2b), indicating *OsFWL5* expression is induced by Cd. Overexpressing *OsFWL5* activates JA-dependent related signaling, promoting us to test JA-signaling related genes expression after Cd treatment. We analyzed *AOS2* and *PR1a* expression and this analysis showed that Cd could induce *AOS2* and *PR1a* expression (Fig. 2c). Cd treatment promotes ROS accumulation in pea plant (Romero-Puertas et al., 2002). These data suggests that Cd might induce plant defense response. To test this inference, we treated *OsFWL5-oe*, *osfwl5* mutants and WT with Cd and inoculated with *Xoo*. Results showed that Cd induced wild type ZH11 resistance to *Xoo* (Fig. 2d). Cd did not further increase *OsFWL5-oe* resistance to *Xoo,* although *OsFWL5-oe* plants accumulated more *AOS2* and *PR1a* transcripts relative to wild type after Cd induction (Fig. 2d; 2e). One of the possible reasons is that *OsFWL5-oe* plants show high resistance to *Xoo* with the lesion length less than 0.5 cm. Cd induced resistance, *AOS2* and *PR1a* expression was impaired in *osfwl5* mutants (Fig. 2d; 2f). These results suggested that *OsFWL5* is required for Cd-induced defense response.

The amino acid sequence of OsFWL5 from ZH11 is identical to that from another geng/japonica variety Nipponbare (Additional file 1: Figure S4). The sequence diversity of OsFWL5 from gene/japonica-type accessions and jing/indica-type accessions is correlated with Zn content in both rice and yeast cells, while yeast cells accumulate similar Cd concentrations expressing both types of OsFWL5 (Song et al., 2015). In this study, OsFWL5 mediated rice defense may be associated with Cd, suggesting that OsFWL5 from jing/indica-type accessions might also play a role in rice resistance. Further studies are needed to provide insight on this perspective.

In conclusion, in this study we have confirmed the novel function of rice *OsFWL5*. Activation of *OsFWL5* expression in rice triggers H_2_O_2_ accumulation and cell death. We further demonstrated that *OsFWL5* positively regulates PTI response and disease resistance. In addition, *OsFWL5* is required for Cd-induced defense response. The grains of *OsFWL5-*oeverexpressing rice accumulated more beneficial element Zn and less toxic element Cd content (Song et al., 2015). So breeders can use *OsFWL5* for rice genetic improvement through screening alleles with optimal expression level.

## Additional files

Additional file 1: **Figure S1**. Expression patterns of *OsFWL5* in rice susceptible and resistant reactions. **Figure S2.** *osfwl5* genotype characterization. **Figure S3.** Performance of *osfwl5* plants. **Figure S4.** Comparison of OsFWL5 amino acid sequences. (PPTX 103 kb)

Additional file 2: **Table S1.** PCR primers used for construction of vectors, detection of positive transgenic plants, mutant analysis, and sequencing. **Table S2.** Primers used for quantitative PCR in gene expression analysis. (DOC 43 kb)

Additional file 3: Materials and Methods. (DOC 37 kb)

## Data Availability

All data generated or analyzed during this study are included in this published article [and its supplementary information files].

## Abbreviations

AOS2: allene oxide synthase 2
*FWL*: *fruit-weight 2.2-like*
ICS1: isochorismate synthase 1
JA: jasmonic acid
JAZ: jasmonate ZIM-domain protein
PR1a: acidic pathogenesis-related protein 1
PTI: pathogen-associated molecular pattern (PAMP)-triggered immunity
ROS: reactive oxygen species
SA: salicylic acid
*Xoo*: *Xanthomonas oryzae* pv. *oryzae*
