# Supplementary material for: Overexpression a “fruit-weight 2.2-like” gene OsFWL5 improves rice resistance
Source: Rice (N Y). 2019 Jul 16;12:51. doi: 10.1186/s12284-019-0315-9 (PMC6635517; doi:10.1186/s12284-019-0315-9)
Supplement: Supplementary file 1 — Figure S1. Expression patterns of OsFWL5 in rice susceptible and resistant reactions. Figure S2. osfwl5 genotype characterization. Figure S3. Performance of osfwl5 plants. Figure S4. Comparison of OsFWL5 amino acid sequences. (PPTX 103 kb) [file 12284_2019_315_MOESM1_ESM.pptx]

## Slide 1
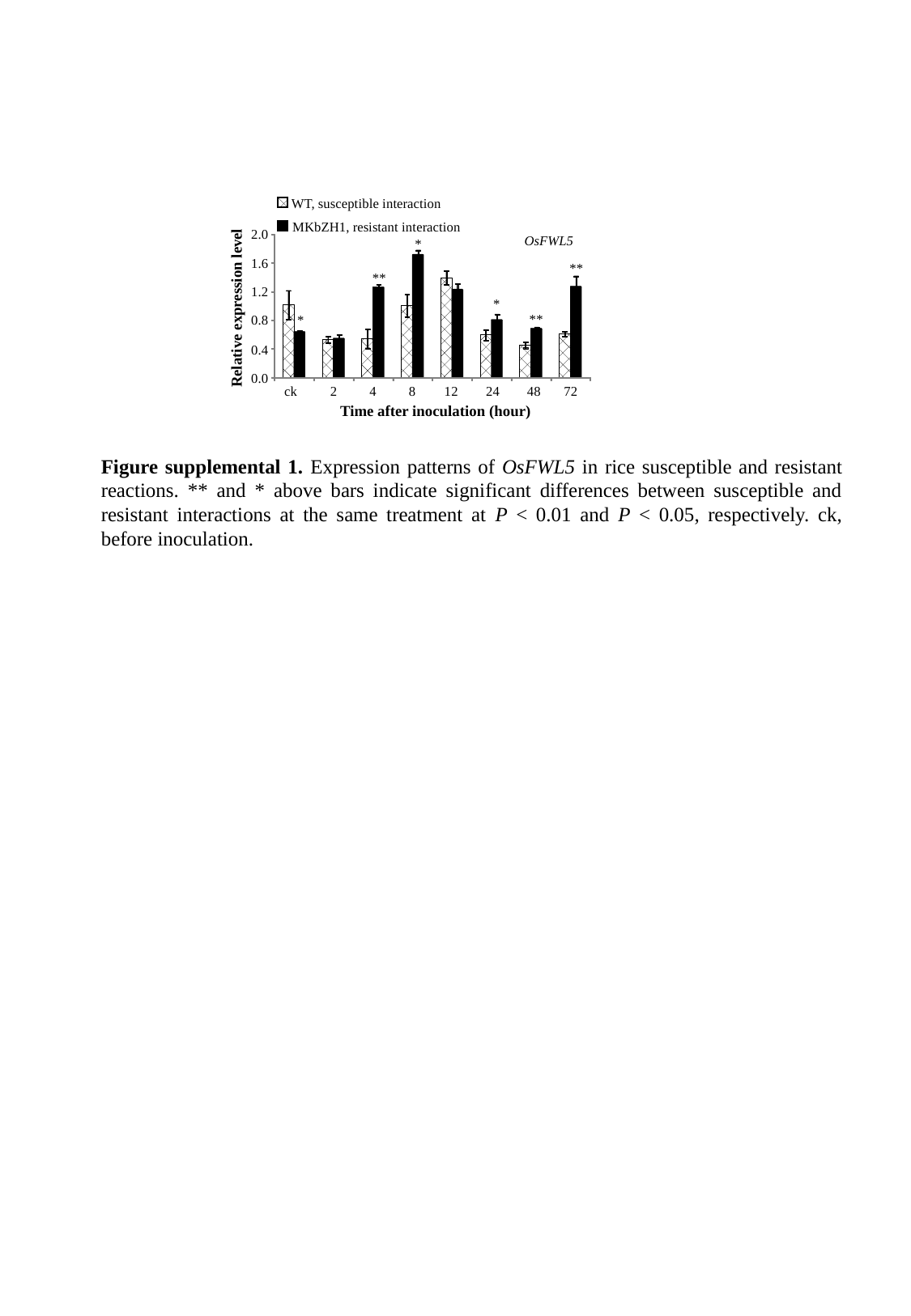

WT, susceptible interaction
MKbZH1, resistant interaction
2.0
OsFWL5
*
1.6
**
**
1.2
*
Relative expression level
**
0.8
*
0.4
0.0
ck
2
4
8
12
24
48
72
Time after inoculation (hour)
Figure supplemental 1. Expression patterns of OsFWL5 in rice susceptible and resistant reactions. ** and * above bars indicate significant differences between susceptible and resistant interactions at the same treatment at P < 0.01 and P < 0.05, respectively. ck, before inoculation.

## Slide 2
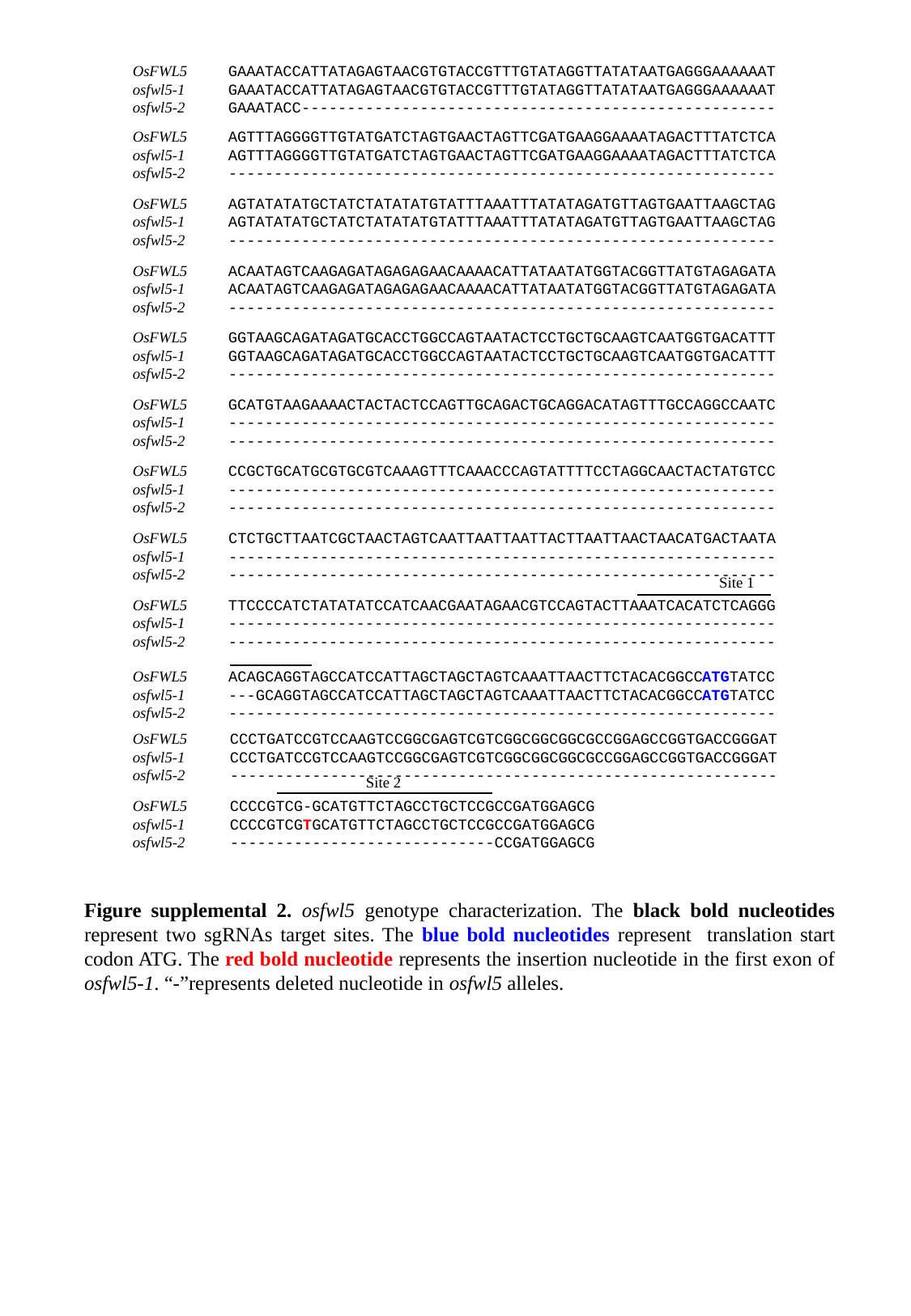

OsFWL5
osfwl5-1
osfwl5-2
GAAATACCATTATAGAGTAACGTGTACCGTTTGTATAGGTTATATAATGAGGGAAAAAAT
GAAATACCATTATAGAGTAACGTGTACCGTTTGTATAGGTTATATAATGAGGGAAAAAAT
GAAATACC----------------------------------------------------
OsFWL5
osfwl5-1
osfwl5-2
AGTTTAGGGGTTGTATGATCTAGTGAACTAGTTCGATGAAGGAAAATAGACTTTATCTCA
AGTTTAGGGGTTGTATGATCTAGTGAACTAGTTCGATGAAGGAAAATAGACTTTATCTCA
------------------------------------------------------------
OsFWL5
osfwl5-1
osfwl5-2
AGTATATATGCTATCTATATATGTATTTAAATTTATATAGATGTTAGTGAATTAAGCTAG
AGTATATATGCTATCTATATATGTATTTAAATTTATATAGATGTTAGTGAATTAAGCTAG
------------------------------------------------------------
OsFWL5
osfwl5-1
osfwl5-2
ACAATAGTCAAGAGATAGAGAGAACAAAACATTATAATATGGTACGGTTATGTAGAGATA
ACAATAGTCAAGAGATAGAGAGAACAAAACATTATAATATGGTACGGTTATGTAGAGATA
------------------------------------------------------------
OsFWL5
osfwl5-1
osfwl5-2
GGTAAGCAGATAGATGCACCTGGCCAGTAATACTCCTGCTGCAAGTCAATGGTGACATTT
GGTAAGCAGATAGATGCACCTGGCCAGTAATACTCCTGCTGCAAGTCAATGGTGACATTT
------------------------------------------------------------
OsFWL5
osfwl5-1
osfwl5-2
GCATGTAAGAAAACTACTACTCCAGTTGCAGACTGCAGGACATAGTTTGCCAGGCCAATC
------------------------------------------------------------
------------------------------------------------------------
OsFWL5
osfwl5-1
osfwl5-2
CCGCTGCATGCGTGCGTCAAAGTTTCAAACCCAGTATTTTCCTAGGCAACTACTATGTCC
------------------------------------------------------------
------------------------------------------------------------
OsFWL5
osfwl5-1
osfwl5-2
CTCTGCTTAATCGCTAACTAGTCAATTAATTAATTACTTAATTAACTAACATGACTAATA
------------------------------------------------------------
------------------------------------------------------------
OsFWL5
osfwl5-1
osfwl5-2
TTCCCCATCTATATATCCATCAACGAATAGAACGTCCAGTACTTAAATCACATCTCAGGG
------------------------------------------------------------
------------------------------------------------------------
OsFWL5
osfwl5-1
osfwl5-2
ACAGCAGGTAGCCATCCATTAGCTAGCTAGTCAAATTAACTTCTACACGGCCATGTATCC
---GCAGGTAGCCATCCATTAGCTAGCTAGTCAAATTAACTTCTACACGGCCATGTATCC
------------------------------------------------------------
OsFWL5
osfwl5-1
osfwl5-2
CCCTGATCCGTCCAAGTCCGGCGAGTCGTCGGCGGCGGCGCCGGAGCCGGTGACCGGGAT
CCCTGATCCGTCCAAGTCCGGCGAGTCGTCGGCGGCGGCGCCGGAGCCGGTGACCGGGAT
------------------------------------------------------------
OsFWL5
osfwl5-1
osfwl5-2
CCCCGTCG-GCATGTTCTAGCCTGCTCCGCCGATGGAGCG
CCCCGTCGTGCATGTTCTAGCCTGCTCCGCCGATGGAGCG
-----------------------------CCGATGGAGCG
Site 1
Site 2
Figure supplemental 2. osfwl5 genotype characterization. The black bold nucleotides represent two sgRNAs target sites. The blue bold nucleotides represent translation start codon ATG. The red bold nucleotide represents the insertion nucleotide in the first exon of osfwl5-1. “-”represents deleted nucleotide in osfwl5 alleles.

## Slide 3
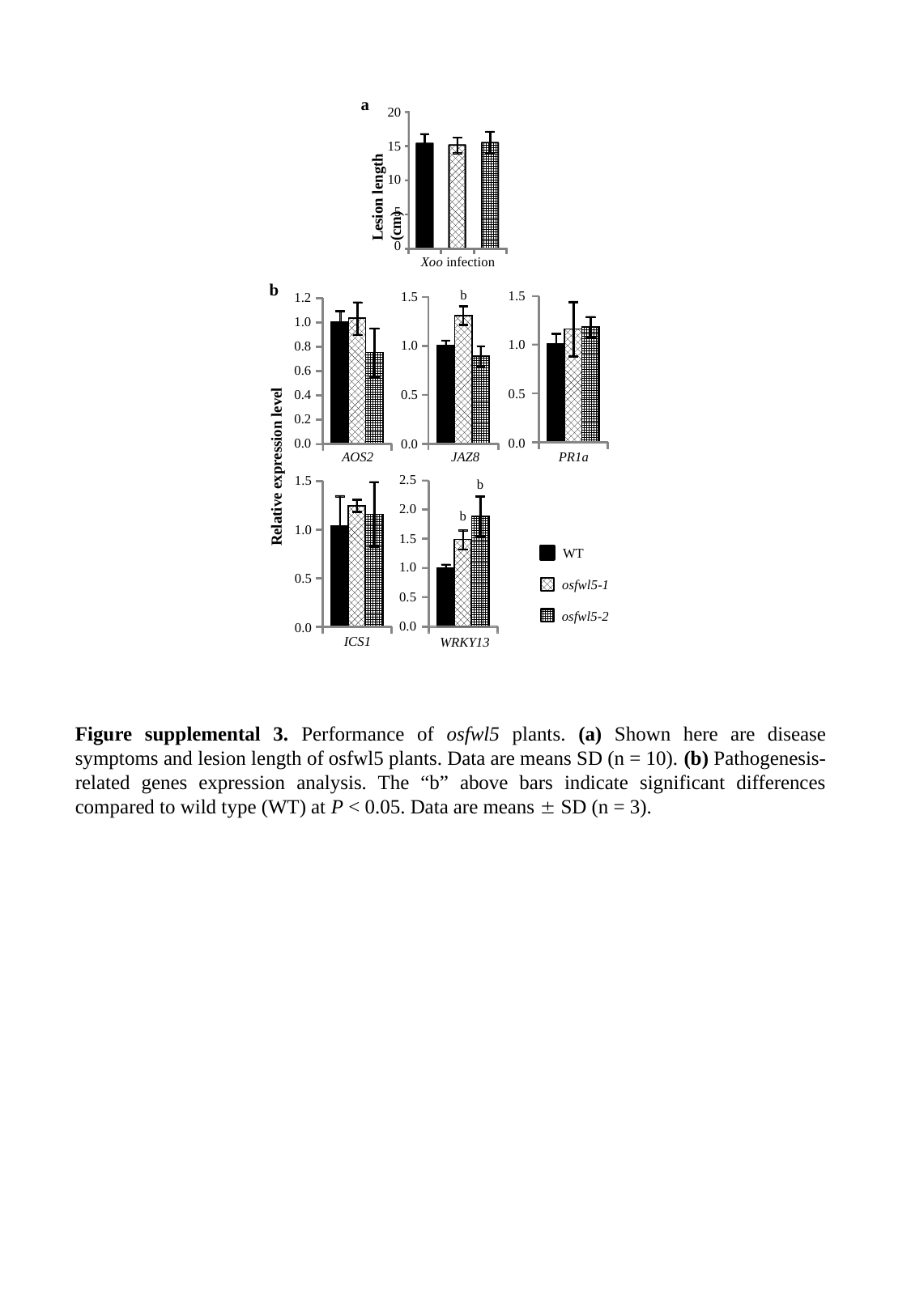

a
20
15
10
Lesion length (cm)
5
0
Xoo infection
b
b
1.5
1.5
1.2
1.0
1.0
1.0
0.8
0.6
0.5
0.5
0.4
0.2
0.0
0.0
0.0
Relative expression level
AOS2
JAZ8
PR1a
2.5
1.5
b
2.0
b
1.0
1.5
WT
1.0
0.5
osfwl5-1
0.5
osfwl5-2
0.0
0.0
ICS1
WRKY13
Figure supplemental 3. Performance of osfwl5 plants. (a) Shown here are disease symptoms and lesion length of osfwl5 plants. Data are means SD (n = 10). (b) Pathogenesis-related genes expression analysis. The “b” above bars indicate significant differences compared to wild type (WT) at P < 0.05. Data are means ± SD (n = 3).

## Slide 4
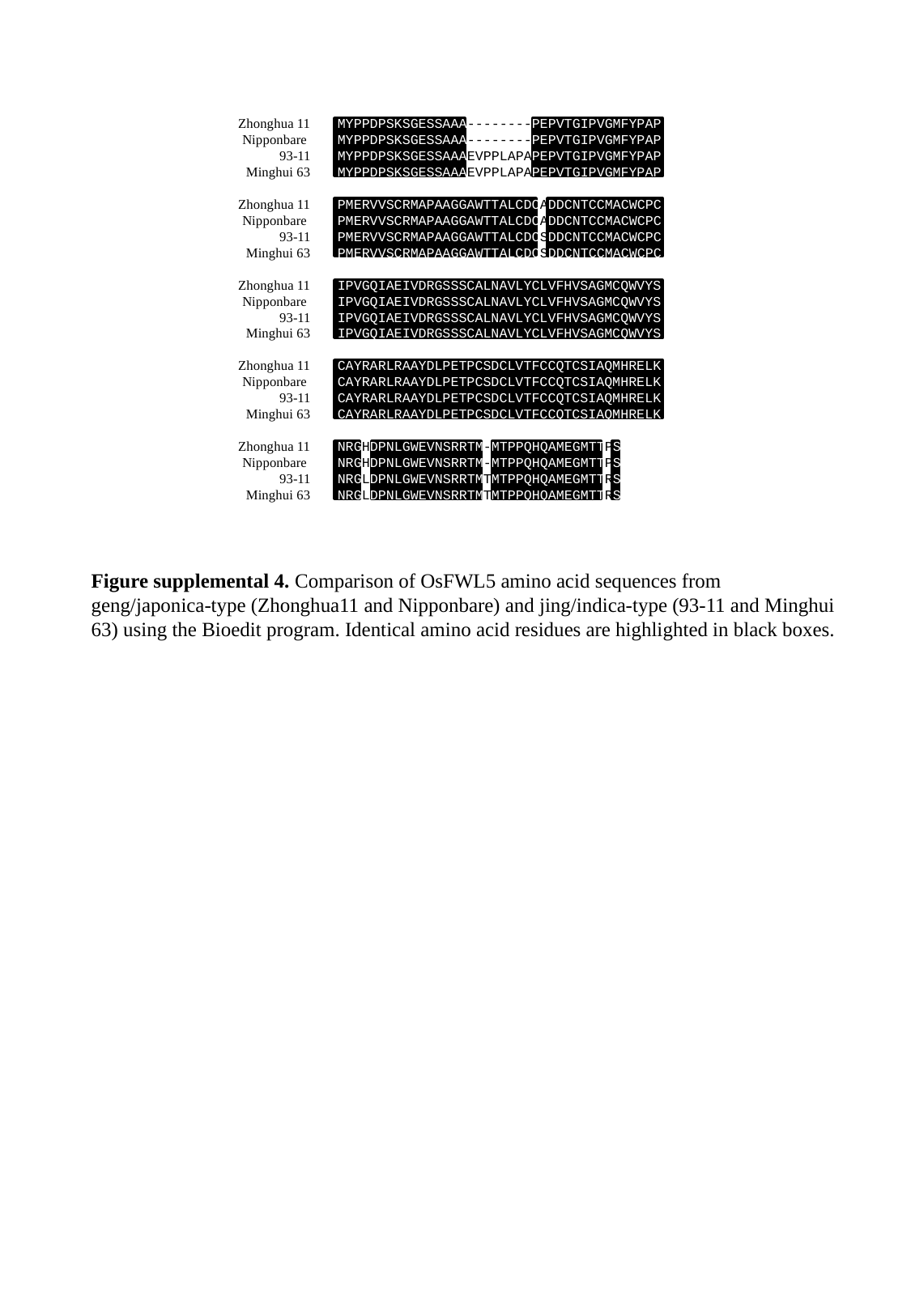

Zhonghua 11
Nipponbare
93-11
Minghui 63
Zhonghua 11
Nipponbare
93-11
Minghui 63
Zhonghua 11
Nipponbare
93-11
Minghui 63
Zhonghua 11
Nipponbare
93-11
Minghui 63
Zhonghua 11
Nipponbare
93-11
Minghui 63
MYPPDPSKSGESSAAA--------PEPVTGIPVGMFYPAP
MYPPDPSKSGESSAAA--------PEPVTGIPVGMFYPAP
MYPPDPSKSGESSAAAEVPPLAPAPEPVTGIPVGMFYPAP
MYPPDPSKSGESSAAAEVPPLAPAPEPVTGIPVGMFYPAP
PMERVVSCRMAPAAGGAWTTALCDCADDCNTCCMACWCPC
PMERVVSCRMAPAAGGAWTTALCDCADDCNTCCMACWCPC
PMERVVSCRMAPAAGGAWTTALCDCSDDCNTCCMACWCPC
PMERVVSCRMAPAAGGAWTTALCDCSDDCNTCCMACWCPC
IPVGQIAEIVDRGSSSCALNAVLYCLVFHVSAGMCQWVYS
IPVGQIAEIVDRGSSSCALNAVLYCLVFHVSAGMCQWVYS
IPVGQIAEIVDRGSSSCALNAVLYCLVFHVSAGMCQWVYS
IPVGQIAEIVDRGSSSCALNAVLYCLVFHVSAGMCQWVYS
CAYRARLRAAYDLPETPCSDCLVTFCCQTCSIAQMHRELK
CAYRARLRAAYDLPETPCSDCLVTFCCQTCSIAQMHRELK
CAYRARLRAAYDLPETPCSDCLVTFCCQTCSIAQMHRELK
CAYRARLRAAYDLPETPCSDCLVTFCCQTCSIAQMHRELK
NRGHDPNLGWEVNSRRTM-MTPPQHQAMEGMTTPS
NRGHDPNLGWEVNSRRTM-MTPPQHQAMEGMTTPS
NRGLDPNLGWEVNSRRTMTMTPPQHQAMEGMTTRS
NRGLDPNLGWEVNSRRTMTMTPPQHQAMEGMTTRS
Figure supplemental 4. Comparison of OsFWL5 amino acid sequences from geng/japonica-type (Zhonghua11 and Nipponbare) and jing/indica-type (93-11 and Minghui 63) using the Bioedit program. Identical amino acid residues are highlighted in black boxes.
