# Supplementary material for: Overexpression a “fruit-weight 2.2-like” gene OsFWL5 improves rice resistance
Source: Rice (N Y). 2019 Jul 16;12:51. doi: 10.1186/s12284-019-0315-9 (PMC6635517; doi:10.1186/s12284-019-0315-9)
Supplement: Supplementary file 2 — Table S1. PCR primers used for construction of vectors, detection of positive transgenic plants, mutant analysis, and sequencing. Table S2. Primers used for quantitative PCR in gene expression analysis. (DOC 43 kb) [file 12284_2019_315_MOESM2_ESM.doc]

**Table S1.** PCR primers used for construction of vectors, detection of positive transgenic plants, mutant analysis, and sequencing

| Primer name | Forward primer (5’-3’) | Reverse primer (5’-3’) | Use |
| --- | --- | --- | --- |
| OsFWL5-F/R | ATGTATCCCCCTGATCCGTC | TTATGAGGGAGTTGTCATTCCTT | construction of *OsFWL5*-over- expression vector |
| GUS2F/R | CCAGGCAGTTTTAACGATCAGTTCGC | GAGTGAAGATCCCTTTCTTGTTACCG | Detection of positive transgenic plants |
| TEV | CGAATCTCAATCAAGCAT |  | Detection of positive transgenic plants |
| OsFWL5-3F/3R | AATCACATCTCAGGGACAGCGTT TTAGAGCTAGAAATAGCAAGTTA | GCTGTCCCTGAGATGTGATTG CCACGGATCATCTGCACAAC | Construction of *OsFWL5*-CRISPR vector |
| OsFWL5-12F/12R | AGCAGGGTAGAACATGCCGAGTT TTAGAGCTAGAAATAGCAAGTTA | TCGGCATGTTCTACCCTGCTG CCACGGATCATCTGCACAAC | Construction of *OsFWL5*-CRISPR vector |
| OsU3-NK-F/SX-R | AGCGGTACCAGTCCAGCTAGCGTAATTCATCCAGGTCTCCAAGTTCTAG | TGCGAGCTCATCCCATCTAGAGCTGTGCCGTACGACGGTACGAGGTAC | Construction of *OsFWL5*-CRISPR vector |
| pCXUN-F1/R1 | AGGCACCCCAGGCTTTACAC | ACTAGATCGGGAATTCACTGGC | Detection of positive transgenic plants |
| OsFWL5-TF1/TR1 | GCACCAGCACGCCATACAAC | GTGGGTCCTTCCGAGATTTA | Analysis of *osfwl5* genotype |

**Table S2. Primers used for quantitative PCR in gene expression analysis**

| Primer name | Forward primer (5’-3’) | Reverse primer (5’-3’) |
| --- | --- | --- |
| FWL5RTF2/R2 | CACCGTGAGCTCAAGAACC | GGGAGTTGTCATTCCTTCCA |
| Actin-F/R | TGTATGCCAGTGGTCGTACCA | CCAGCAAGGTCGAGACGAA |
| ICS1-F/R | TATGGTGCTATCCGCTTCGAT | CGAGAACCGAGCTCTCTTCAA |
| PR1a-F/R | CGTCTTCATCACCTGCAACTACTC | CATGCATAAACACGTAGCATAGCA |
| AOS2-F/R | CAATACGTGTACTGGTCGAATGG | AAGGTGTCGTACCGGAGGAA |
| OsWRKY13RTF/R | TTTGGGAAAGCGTTGATTAGT | GCGCACACACTCCAACTC |
| OsNAC4-RTF/R | TCCTGCCACCATTCTGAGATG | TTGCAGAATCATGCTTGCCAG |
| JAZ8-RTF/R | GAAGGCTCAACAGCTGACCAT | TTGGTGGACGGGAAGTTCTC |
