# Supplementary material for: Overexpression a “fruit-weight 2.2-like” gene OsFWL5 improves rice resistance
Source: Rice (N Y). 2019 Jul 16;12:51. doi: 10.1186/s12284-019-0315-9 (PMC6635517; doi:10.1186/s12284-019-0315-9)
Supplement: Supplementary file 3 — Materials and Methods. (DOC 37 kb) [file 12284_2019_315_MOESM3_ESM.doc]

**Vector construction and rice transformation**

For overexpression of *OsFWL5, OsFWL5* full length cDNA which were amplified from japonica/geng variety Zhonghua 11 (ZH11) leaf cDNA was inserted into vector pU1301 with gene specific primers (Table S1). *OsFWL5*-knockout mutants *osfwl5* were generated with two sgRNAs designed in 5’ UTR and the first exon under the control of rice *OsU3* and *OsU6a* promoters, respectively (Table S1) (Ma et al., 2015). The recombined vectors were respectively transformed into *Agrobacterium tumefaciens* strain EHA105 by electroporation. *Agrobacterium*-mediated transformation was performed using calli derived from embryo of wild type ZH11 (Lin and Zhang 2005).

**Cd treatment**

Rice plants growing in greenhouse until the booting stage were transferred in culture solution with 100 μM CdCl_2_ and in culture solution without CdCl_2_ to serve as control (mock treatment). The plants were treated for 2 days, and then inoculated with *Xoo* strain PXO341.

**Pathogen inoculation**

To evaluate bacterial blight disease, rice plants were inoculated with Philippine *Xoo* strain PXO341 using the leaf-clipping method at the booting stage (Ke et al., 2017). Sample were collected at represented time point. Disease was scored by measuring lesion length at about 14 days after inoculation. The bacterial growth rate in rice leaves was measured by counting the colony forming units (Ke et al., 2017).

**Gene expression analysis**

Quantitative reverse transcription (qRT)-PCR was conducted using gene-specific primers (Table S2) as described previously (Qiu et al., 2007). The expression level of the rice actin gene was used to standardize the RNA sample amount for each qRT-PCR. The expression level relative to control was presented.

**Sequence alignment**

ZH11 *OsFWL5* full length cDNA was amplified from ZH11 leaf cDNA and sequenced, and amino acid sequence was predicted. The cDNA sequence of *OsFWL5* from ZH11 is identical to that from Nipponbare (LOC_Os10g02300*,* http://rice.plantbiology.msu.edu/index.shtml). The accession numbers of OsFWL5 in RIGW ([http://rice.hzau.edu.cn](http://rice.hzau.edu.cn/)) are LOC_Os10g02300 (Nipponbare), MH10t0020100-02 (Minghui 63), and BGIOSGA032386 (93-11). The amino acid sequences were aligned using BioEdit (<https://bioedit.software.informer.com/>).

**Statistical analyses**

The statistical significance of differences between control and sample treatments were assessed using the pair-wise *t*-test installed in the Microsoft Office Excel program.

**Reference**

Ke Y, Hui S, M Yuan (2017) *Xanthomonas oryzae* pv. *oryzae* Inoculation and Growth Rate on Rice by Leaf Clipping Method, *Bio-Protocol* 7 19.

Lin YJ, Zhang Q (2005) Optimising the tissue culture conditions for high efficiency transformation of indica rice. Plant Cell Rep. 23:540-547.

Ma X, Zhang Q, Zhu Q, Liu W, Chen Y, Qiu R, Wang B, Yang Z, Li H, Lin Y, Xie Y, Shen R, Chen S, Wang Z, Chen Y, Guo J, Chen L, Zhao X, Dong Z, Liu YG (2015) A Robust CRISPR/Cas9 System for Convenient, High-Efficiency Multiplex Genome Editing in Monocot and Dicot Plants. Mol Plant 8:1274-1284.

Qiu D, Xiao J, Ding X, Xiong M, Cai M, Cao Y, Li X, Xu C, Wang S (2007) OsWRKY13 mediates rice disease resistance by regulating defense-related genes in salicylate- and jasmonate-dependent signaling. Mol Plant Microbe Interact. 20:492-499.
